# Infection responsive coatings to reduce biofilm formation and encrustation of urinary catheters

**DOI:** 10.1093/jambio/lxad121

**Published:** 2023-06-09

**Authors:** Anthony J Slate, Ocean E Clarke, Mina Kerio, Jonathan Nzakizwanayo, Bhavik Anil Patel, Brian V Jones

**Affiliations:** Department of Life Sciences, University of Bath, Claverton Down, Bath BA2 7AY, United Kingdom; Department of Life Sciences, University of Bath, Claverton Down, Bath BA2 7AY, United Kingdom; School of Applied Sciences, University of Brighton, Brighton BN2 4GJ, United Kingdom; Department of Life Sciences, University of Bath, Claverton Down, Bath BA2 7AY, United Kingdom; School of Applied Sciences, University of Brighton, Brighton BN2 4GJ, United Kingdom; Department of Life Sciences, University of Bath, Claverton Down, Bath BA2 7AY, United Kingdom

**Keywords:** catheter-associated urinary tract infection, crystalline biofilm, *Proteus mirabilis*, theranostic, infection responsive coating

## Abstract

**Aims:**

The care of patients undergoing long-term urethral catheterization is frequently complicated by *Proteus mirabilis* infection. This organism forms dense, crystalline biofilms, which block catheters leading to serious clinical conditions. However, there are currently no truly effective approaches to control this problem. Here, we describe the development of a novel theranostic catheter coating, to simultaneously provide early warning of blockage, and actively delay crystalline biofilm formation.

**Methods and Results:**

The coating comprises of a pH sensitive upper polymer layer (poly(methyl methacrylate-co-methacrylic acid); Eudragit S 100^®^) and a hydrogel base layer of poly(vinyl alcohol), which is loaded with therapeutic agents (acetohydroxamic acid or ciprofloxacin hydrochloride) and a fluorescent dye, 5(6)-carboxyfluorescein (CF). The elevation of urinary pH due to *P. mirabilis* urease activity results in the dissolution of the upper layer and release of cargo agents contained in the base layer. Experiments using *in vitro* models, which were representative of *P. mirabilis* catheter-associated urinary tract infections, demonstrated that these coatings significantly delay time taken for catheters to block. Coatings containing both CF dye and ciprofloxacin HCl were able to provide an average of *ca*. 79 h advanced warning of blockage and extend catheter lifespan *ca*. 3.40-fold.

**Conclusions:**

This study has demonstrated the potential for theranostic, infection-responsive coatings to form a promising approach to combat catheter encrustation and actively delay blockage.

Significance and Impact of Study This study builds on previous research conducted in our group and further optimizes the proposed, infection responsive, urinary catheter coating. For the first time, therapeutic agents have been incorporated into this theranostic coating design. This resulted in an advanced warning of blockage (*e.g.*, dye release) and actively delays catheter blockage times (*e.g.*, therapeutic agent release into the bladder). Such coatings represent a major advance in the treatment of catheter-associated urinary tract infections (CAUTIs).

## Introduction

Indwelling urinary catheters are deployed in a wide variety of circumstances related to the management of bladder dysfunction, as well as the management and monitoring of urinary output (Stickler, 2008). The duration of catheterization can range from short-term use in clinical settings (up to 7 days), to long-term use (> 28 days) in patients cared for in the community (Stickler, 2008; Loveday *et al*., 2014; Stickler, 2014; Shackley *et al*., 2017). In keeping with the prolific use of these devices, catheter-associated urinary tract infections (CAUTIs) continue to be among the most prevalent healthcare-associated infections in both hospital and community care settings; with up to 80 % of all healthcare-associated urinary tract infections (UTIs) related to the use of indwelling catheters (Parker *et al*., 2017; Clark and Wright, 2019; Yakusheva *et al*., 2019). Even with the most scrupulous nursing care and rigorous application of sterile closed drainage systems, bacteriuria is almost inevitable in patients undergoing long-term catheterization (> 28 days), and this group of patients are particularly vulnerable to resulting complications (Nicolle, 2014).

In particular, the encrustation and blockage of catheters is a common and serious complication, which may be experienced by up to 50 % of individuals undergoing long-term catheterization (Nicolle, 2014). If undetected, blockage can lead to the reflux of infected urine to the upper urinary tract, which may subsequently result in pyelonephritis, septicaemia, and endotoxic shock (Stickler and Zimakoff, 1994; Jordan *et al*., 2015). *Proteus mirabilis* is a common pathogen of the catheterized urinary tract, associated with up to 44 % of CAUTIs, and is the primary cause of catheter blockage (O'Hara *et al*., 2000; Jacobsen *et al*., 2008; Nicolle, 2014). This organism forms dense crystalline biofilms, which encrust catheters and occlude urine flow (Griffith *et al*., 1976; Cox and Hukins, 1989; Clapham *et al*., 1990; Dumanski *et al*., 1994; Stickler and Zimakoff, 1994; Morris *et al*., 1999, Jones *et al*., 2005). This process is driven by the production of a potent urease enzyme by *P. mirabilis*, which generates ammonia through the hydrolysis of urea present in urine, elevating urinary pH (Griffith *et al*., 1976). Under these alkaline conditions, the precipitation of calcium phosphate (apatite) and magnesium ammonium phosphate (struvite) occurs, producing microcrystalline aggregates that become incorporated within developing *P. mirabilis* biofilms (Hedelin *et al*., 1984; Cox and Hukins, 1989). Integration of crystal aggregates into biofilms further stabilizes and enhances their growth leading to the mineralization of biofilms, the formation of crystalline biofilm structures, and the eventual blockage of catheters (Griffith *et al*., 1976; Cox and Hukins, 1989; Clapham *et al*., 1990; Dumanski *et al*., 1994; Stickler and Zimakoff, 1994; Morris *et al*., 1999; Jones *et al*., 2005).

Despite the potentially severe consequences for patients, catheter blockage is typically not detected until more serious conditions arise. The initial bacterial colonization of the catheterized urinary tract is usually asymptomatic, and blockage can occur rapidly with little warning (Kohler‐Ockmore and Feneley, 1996; Tambyah and Maki, 2000; Stickler *et al*., 2006; Long *et al*., 2014; Stickler, 2014). This is particularly problematic for individuals cared for in the community where continual clinical surveillance is not feasible. Although a range of catheters with antimicrobial coatings are available, their ability to control infection during even short-term catheterization is poor, and currently all available catheter types are vulnerable to encrustation (Stickler, 2014; Nzakizwanayo *et al*., 2016; Cortese *et al*., 2018). Therefore, novel approaches, which provide advanced warning, or prevent infection and catheter blockage, are clearly needed and would considerably improve patient wellbeing.

**Figure 1.**
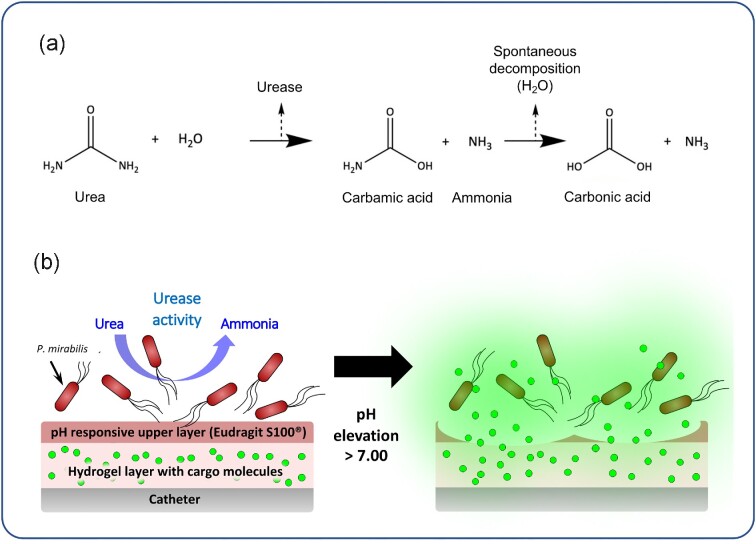
**Infection responsive coating design and function. (a)** Schematic showing urease-mediated hydrolysis of urea to generate ammonia, which elevates urinary pH during *P. mirabilis* infection. **(b)** Overview of coating design and response to *P. mirabilis* infection. Elevation of urinary pH driven by *P. mirabilis* urease activity leads to dissolution of the upper Eudragit S 100^®^ ‘trigger’ layer and release of cargo molecules contained in the lower hydrogel layer.

To address this, we have recently developed a prototype infection-responsive catheter coating that can provide early warning of catheter blockage (Milo *et al*., 2016; Milo *et al*., 2017). This coating comprised a pH sensitive outer layer (Eudragit S 100 ^®^), which sealed a lower hydrogel basement layer (poly(vinyl alcohol); PVA) containing the self-quenching fluorescent dye 5(6)-carboxyfluorescein. The elevation of urinary pH by *P. mirabilis* results in degradation of the upper Eudragit S 100 ^®^ layer and release of the fluorescent dye to give advanced warning of the potential for catheter blockage ( Figure. 1). Here, we expand on these initial studies, describing the further development of this coating technology to include delivery of therapeutic agents to actively delay catheter blockage. Evaluation of these novel coatings in representative models of CAUTI demonstrated the potential for these to provide a theranostic approach for the control of catheter blockage, by simultaneously treating infection (therapeutic) while providing advanced warning of catheter blockage (diagnostic).

## Materials and methods

### Bacterial strains, media, and routine culture

*Proteus mirabilis* clinical strain RS1 (Royal Sussex County Hospital) was used throughout this study. All chemicals, reagents, and growth media were obtained from Fisher Scientific (UK), Merck (UK), and Oxoid (UK), unless otherwise stated. Bacteria were routinely cultured in Lysogeny Broth [LB (Miller): 5 g L^−1^ yeast extract, 10 g L^−1^ tryptone, 10 g L^−1^ sodium chloride] at 37 °C with shaking for 18 h; for the isolation of single colonies of *P. mirabilis* and the suppression of swarming motility, strains were grown on no salt-LB (NSLB) agar (5 g L^−1^ yeast extract, 10 g L^−1^ tryptone, 20 g L^−1^ agar).

### Catheter coating protocol

Catheter coating production was adapted from protocols originally described by Milo *et al*., (2016), and coatings were applied to the external surface of the catheter from the catheter tip to the start of the retention balloon.

#### Salinization

The catheter surface was modified to promote the attachment of the hydrogel/polymer coating. This salinization process covered the surface with organofunctional alkoxysilane molecules, which increased the hydrophobicity of the catheter surface (forming covalent -Si-O-Si- bonds). The tips of the catheters were submerged in a solution of ammonia hydroxide (33 % v/v) and hydrogen peroxide (30 % v/v) (at a 1:1 ratio) for 30 minutes with sonication. The catheters were rinsed in sterile deionized water (thrice) and dried under a constant stream of nitrogen. The catheter tips were placed in 1 % v/v (3-aminopropyl)triethoxysilane (APTES) in dimethylformamide overnight (at room temperature). The catheters were washed three times in sterile deionized water and dried under a constant stream of nitrogen.

#### PVA Preparation

High molecular weight poly(vinyl alcohol) (PVA; 146,000 – 186,000g mol^−1^) was produced at 30 % w/v in sterile deionized water. This was heated to 97 °C with constant aggregation for 10 minutes. Once dissolved and cooled, the therapeutic payload was added at a 1:1 ratio. The final PVA concentration was 15 % w/v. If 5(6)-carboxyfluorescein (CF) was utilized this was added at a final concentration of 100 mM, whilst acetohydroxamic acid (AHA) and ciprofloxacin hydrochloride were incorporated at 5 mg mL^−1^.

#### Preparation of Eudragit S 100^®^

The pH sensitive, Eudragit S 100^®^ polymer was prepared by combining, 109.1 mL acetone, 163.5 mL isopropanol, and 10.7 mL deionized water, this solution was then aliquoted into two equal volumes (141.65 mL). Eudragit polymer (15.6 g) was added to one of the diluent mixtures and homogenized at room temperature. To the other half of the diluent mixture, hydrated magnesium silicate (7.81 g, talc - an anti-tacking agent) and triethyl citrate (1.4 mL) were added. This was agitated for 10 minutes using a high shear mixer ( 1,000rpm). The solutions were then combined (slowly with constant agitation) and this was filtered (Grade 1, 85 mm; Whatman, UK) using a Büchner filter under vacuum to remove the hydrated magnesium silicate. This step was repeated three times. The polymer solution was stored at 4 °C until required.

#### Catheter coating

Foley urinary catheters (BARDIA^®^ AQUAFIL^®^ All Silicone Foley Catheter; Bard Limited, UK) were coated between the balloon and the tip (whilst deflated) with 1 mL of the prepared 15 % w/v PVA containing the therapeutic payload. The catheter eyelet was obstructed, during the coating process, to inhibit polymer blockage. This was then stored at − 20 °C overnight to promote cryogenic crosslinking. The catheters were thawed at room temperature for 4 h before being dip-coated with the secondary pH responsive polymer Eudragit S 100^®^. The dip coating procedure was carried out manually, with the catheters being vertically submerged into the polymer for 3 seconds followed by a 5-minute solvent evaporation time, to ensure full Eudragit S 100^®^ coverage, this process was repeated 20 times. The catheters were dried at 4 °C overnight. After 18 h, the barrier blocking the eyelet was replaced and the catheters were coated using the aforementioned process a further 10 times. Once coated, the catheters were stored at 4 °C, overnight. This process was repeated once more. Following this, the catheter eyelet was unblocked and Eudragit S 100^®^ was added a further five times to seal the PVA layer around the eyelet (total Eudragit S 100^®^ coatings: 45). This coating ensured the therapeutic payload below was adequately sealed prior to experimentation. The catheters were then stored at 4 °C until required.

### Growth curves

In a 96-well plate, wells containing 100 µL of tryptone soya broth (TSB) or TSB supplemented with 100 mM 5(6)-carboxyfluorescein were inoculated with 10^5^ CFU mL^−1^ of *P. mirabilis* strain RS1 from a mid-log phase culture. Uninoculated wells served as negative controls. The plate was then incubated in a plate reader (Multiskan™ FC Microplate Reader) at 37 °C while shaking, and optical density (OD_600 nm_) was measured at 1 hour intervals over 24 hours. Data from three biological replicates (*n* = 3) were pooled to create a mean overall growth curve.

### Artificial urine

The artificial urine (AU) medium used throughout this study has previously been described (Nzakizwanayo *et al*., 2019). Briefly, the AU was initially prepared as a 5 × concentrated stock solution containing; sodium disulfate (11.5 g L^−1^), magnesium chloride (hexahydrate) (3.25 g L^−1^), sodium chloride (23 g L^−1^), trisodium citrate (3.25 g L^−1^), sodium oxalate (0.1 g L^−1^), potassium dihydrogen orthophosphate (14 g L^−1^), potassium chloride (8 g L^−1^), ammonium chloride (5 g L^−1^), gelatine (25 g L^−1^), and tryptone soya broth (5 g L^−1^). Stock solutions of urea (125 g) and calcium chloride dihydrate (3.25 g) were sterilized separately in 400 mL sterile deionized water using a Nalgene Vacuum Filter System (0.44 μm nitrocellulose membrane; Sartorius, UK), while other components were sterilized by autoclaving. For use in bladder models, all components were combined and diluted to 1 × strength using sterile deionized water (3.6 L), the final pH was adjusted to 6.1 prior to experimentation.

### *In vitro* models of the catheterized urinary tract

Bladder models were conducted as previously described (Nzakizwanayo *et al*., 2019). The bladder models consisted of a double-walled glass chamber (50 mm internal chamber diameter) maintained at 37 °C by a water jacket supplied from a circulating water bath. All silicone foley catheters (BARDIA^®^ AQUAFIL^®^; Bard Limited, UK) which were either uncoated or coated with the aforementioned polymer design were inserted into the 'bladder' and retention balloons inflated with 10 mL sterile saline. The catheter was then attached to a drainage bag to form a sterile, closed drainage system. Artificial urine was supplied to the 'bladder' at a constant flow rate of *ca*. 0.75 mL minute^−1^ (calculated individually for each model). The *in vitro* bladder model systems were inoculated with 10 mL of a *P. mirabilis* culture containing ∼10^9^ CFU mL^−1^ and bacterial cells were allowed to establish within the model for 1 h before flow was activated. The number of viable cells in the residual urine and the pH of the medium were measured at the start of the experiment and following catheter blockage. The pH was also measured at 4 h to determine the effect the coating had on the AU.

### Scanning electron microscopy (SEM)

To visualize catheter encrustation and coating structure/dissolution SEM was conducted with minimal sample preparation as per Holling *et al*., (2014). Coated Foley catheters were removed from the *in vitro bladder* models after 10 h incubation. Samples were visualized after a 10 h incubation period as this allowed all coatings (and controls) to be visualized prior to blockage, but after a significant time of elevated pH, in order to determine the extent of catheter coating degradation. Catheter sections were recovered for visualization by making initial incisions through the coating directly below the eyelet, and a second distal incision to remove a 1 cm section. Samples were mounted on aluminium stubs and placed in a desiccator containing silica gel beads (2 - 5mm), overnight. SEM was conducted using a Hitachi SU3900 large chamber, variable pressure SEM (Hitachi High-Tech Corporation, Japan).

### Statistical analysis

Statistical analysis was conducted by performing one-way or two-way ANOVAs as appropriate, coupled with Dunnett’s correction for multiple comparisons, using GraphPad Prism (version 8.4.3; GraphPad Software, USA).

## Results

### Impact of optimized coatings on catheter blockage

**Figure 2.**
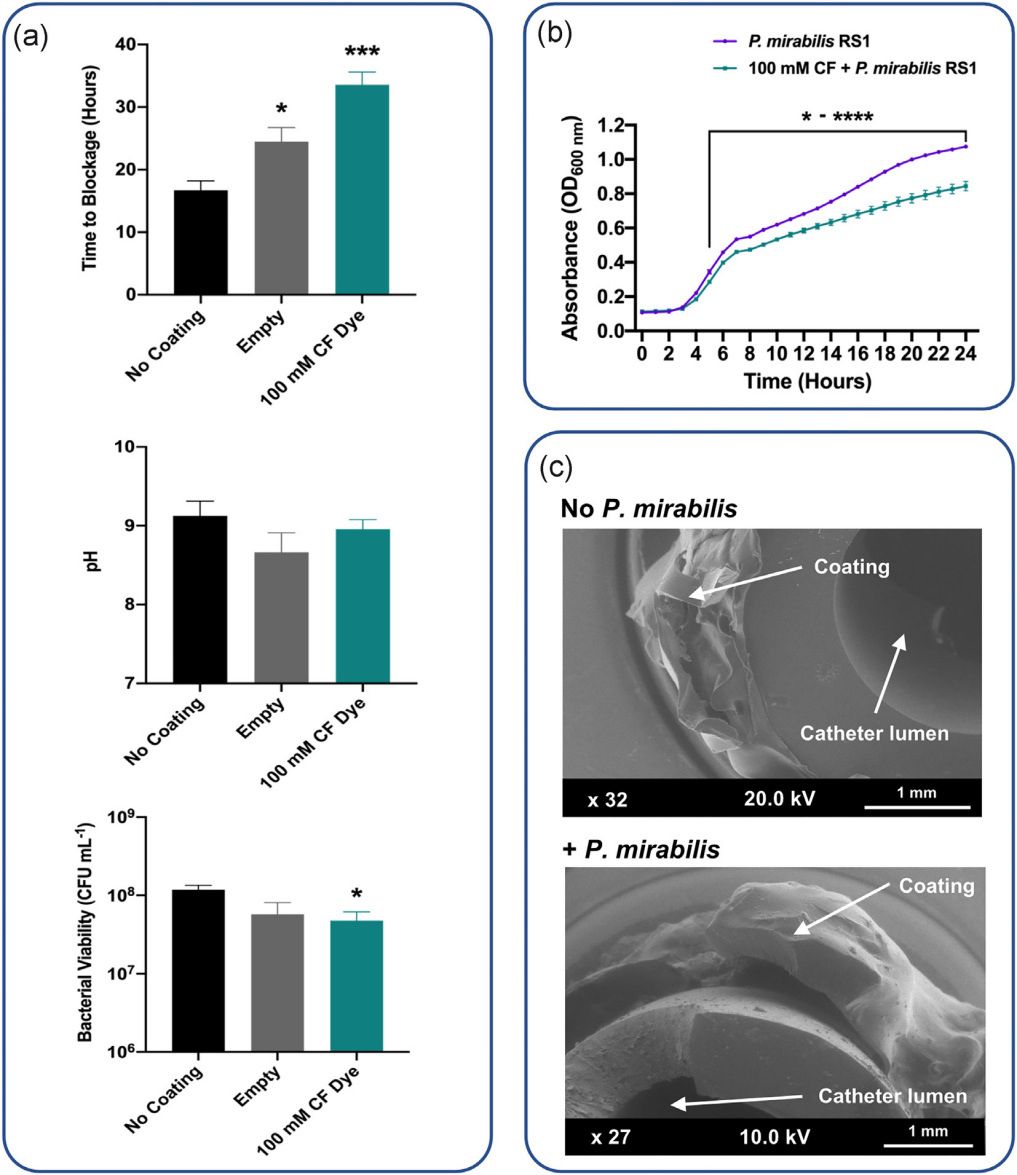
**Effect of optimized coatings on *P. mirabilis* -mediated catheter blockage. (a)** Results of *in vitro* bladder model experiments simulating established infection (10^9^ CFU mL^−1^) showing impact of optimized coatings on catheter blockage, pH elevation, and survival of *P. mirabilis* strain RS1. Measurement of pH and enumeration of remaining viable cells in residual urine was conducted at catheter blockage. **No Coating** = standard All Silicone Foley catheters without coating; **Empty** = All Silicone Foley catheters with coating lacking any cargo molecules in the hydrogel layer; **100 mM CF Dye** = All Silicone Foley catheters with coatings containing 100 mM CF dye in the hydrogel layer. Data represents the mean of five biological replicates ( *n *= 5) and error bars show standard error of the mean, * *P* ≥ .05, ***P ≥ .0001 compared to uncoated catheters. **(b)** Impact of 100 mM 5(6)-carboxyfluorescein on growth of *P. mirabilis* RS1 in TSB. Data represents the mean of three biological replicates and error bars show standard error of the mean. * ^-^ ****P ≥ .05 - .00001. **(c)** Scanning electron micrographs of catheter coatings after 10 h incubation in sterile uninoculated bladder models (No *P. mirabilis* ), or models inoculated with *P. mirabilis* (*+ P. mirabilis* ).

The initial optimization of our coating production involved increasing the proportion of PVA in the hydrogel basement layer and improvements to techniques for application of the upper pH responsive Eudragit S 100 ^®^ layer. To determine the baseline performance of these optimized coatings, we compared the blockage times of catheters with ‘empty’ coatings devoid of cargo molecules, and those containing CF dye only, to standard uncoated All Silicone Foley catheters ( Figure. 2).

In representative models of *P. mirabilis* CAUTI, simulating an established high-level infection (10^9^ CFU mL^−1^), empty coatings alone significantly increased time to blockage by an average of 7.8 h compared with uncoated catheters ( Figure. 2a). The inclusion of 100 mM of 5(6)-carboxyfluorescein in the hydrogel layer of the coating provided further increases in catheter blockage time, on average 16.9 h longer than for uncoated catheters ( Figure. 2a). Coating application did not alter the flow rate of urine through the catheters, the average flow rate of the catheters throughout this study was 0.88 (± 0.012) mL minute^−1^.

The pH of residual urine at the time of catheter blockage was not significantly different in any models with coated catheters compared to uncoated controls ( Figure. 2a). However, a significant reduction in viable cell numbers was observed in models with coatings containing the CF dye ( Figure. 2a), and further investigation revealed a significant impact of the CF dye on *P. mirabilis* growth at concentrations used in the coatings ( Figure. 2b).

Consistent with our previous work (Milo *et al*., 2016), coatings containing CF dye also elicited a visible urine colour change, which was most pronounced ∼ 4 - 5h after activation of models inoculated with *P. mirabilis* , providing, ∼ 29 h advance warning of catheter blockage on average. SEM analysis of catheter sections after 10 h of incubation in the bladder models confirmed stability of coatings in uninoculated control models, and the deterioration of coatings in models infected with *P. mirabilis* ( Figure. 2c).

### Incorporation of therapeutic agents into catheter coatings

To evaluate the ability of our infection responsive coatings to deliver therapeutic agents, we subsequently incorporated either the urease inhibitor acetohydroxamic acid (AHA, marketed commercially as Lithostat^®^), or the antibiotic ciprofloxacin hydrocholoride at 5 mg mL^−1^ into the PVA layer. Release of this amount of each drug into the residual bladder model urine was estimated to generate concentrations well above the indicated therapeutic range for Lithostat^®^ [∼ 30µg mL^−1^; (Mission Pharmacal Company 2023)], and the *P. mirabilis* strain RS1 MIC for ciprofloxacin HCl (0.125 µg mL^−1^). Coating variants containing CF dye in combination with therapeutic agents, as well as those containing therapeutic agents alone, were produced and their impact on *P. mirabilis* crystalline biofilm formation was again evaluated in our infection models.

**Figure 3.**
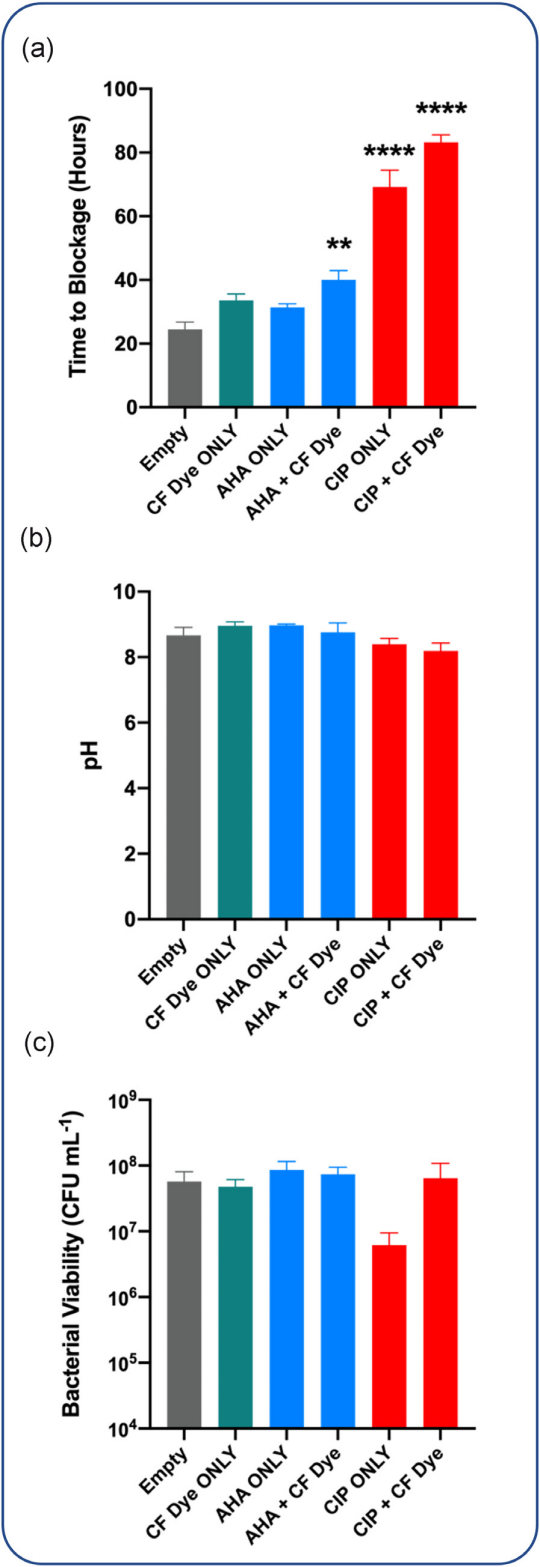
**Impact of theranostic coatings on catheter blockage**. Theranostic coatings containing CF dye, and either the urease inhibitor acetohydroxamic acid or the antibiotic ciprofloxacin HCl, were evaluated using *in vitro* bladder models. **(a)** Time taken for catheters to block; **(b)** pH of residual bladder model urine at time of catheter blockage; and **(c)** viable cell counts in residual urine at time of catheter blockage . **Empty** = All Silicone Foley catheters with coating lacking any cargo molecules in the hydrogel layer; **CF Dye ONLY** = All Silicone Foley catheters with coatings containing 100 mM 5(6)-carboxyfluorescein dye (only) in the hydrogel layer; **AHA** = acetohydroxamic acid (Lithostat^®^) at 5 mg mL^−1^ in the coating; **CIP** = ciprofloxacin at 5 mg mL^−1^ in the coating; and **CF dye** = 5(6)-carboxyfluorescein dye at 100 mM in coating. Data represents the mean of five biological replicates ( *n* = 5) and error bars show standard error of the mean. * *P* ≥ .05, ****P ≥ .00001, compared to empty coatings.

The inclusion of AHA in coatings resulted in no significant increase in time to blockage compared to the empty coating, but incorporation of AHA in combination with CF dye resulted in a nominal but statistically significant increase in blockage time ( Figure. 3a). In contrast, incorporation of ciprofloxacin HCl in coatings alone or in combination with CF dye, produced notable increases in catheter blockage time and increased catheter lifespan by 2.82-fold and 3.40-fold, respectively, compared to the empty coatings ( Figure. 3a). In coatings containing CF dye in conjunction with therapeutic agents, a visual indication of coating activation was also evident after model activation, which provided an average of ∼ 36 h advance warning of blockage for coatings containing AHA and ∼ 79 h for coatings containing ciprofloxacin HCl. No significant differences in urinary pH or viable bacterial counts were observed at time of blockage for any coating evaluated (compared to empty coatings), but viable cells counts in models with ciprofloxacin HCl coatings were lower than for models with ciprofloxacin HCl and CF dye containing coatings ( Figure. 3b and c).

## Discussion

Although a wide range of approaches to control biofilm formation on urethral catheters have been described, and often appear to provide adequate protection against many common species of uropathogenic bacteria, development of effective strategies to control encrustation by *P. mirabilis* poses a particular challenge (Stickler, 2008, 2014; Milo *et al*., 2019; Pelling *et al*., 2019). In this study, our prototype theranostic coatings were able to provide both advanced warning of catheter blockage as well as actively increasing the time taken for *P. mirabilis* to block catheters. While both therapeutic agents tested in our coatings may be administered orally for the treatment of UTIs, localized delivery options have the potential to avoid many of the systemic side effects associated with these drugs and provide a more effective way to combat CAUTIs (Benderev, 1988; Zacchè *et al*., 2015; Wan *et al*., 2018; Marei *et al*., 2021; Moussa *et al*., 2021).

Due to the key role of the *P. mirabilis* urease enzyme in the formation of crystalline biofilms and blockage of urinary catheters, inhibition of this enzyme is an attractive approach to control catheter encrustation by this organism. Urease inhibitors have previously been explored as potential therapeutic treatments to reduce catheter encrustation, and have shown some promise for control of crystalline biofilm formation (Morris and Stickler, 1998; Follmer, 2010). In particular, the structural analogue of urea, AHA is currently marketed under the name Lithostat^®^, and indicated for use in the treatment of UTIs involving urease-producing species and stone formation. However, whilst AHA has demonstrated effective inhibition of urolithiasis in some clinical trials, it has severe side effects for many patients when taken orally. These include headaches, gastrointestinal upset, thrombophlebitis, and dermatitis, which limit its clinical application; it is currently only recommended as a treatment of last resort (Williams *et al*., 1984; Griffith *et al*., 1988; Georgi, 2004; York *et al*., 2015; Zisman, 2017; Abou Chakra *et al*., 2020).

However, in our experiments, delivery of AHA *via* our coating system did not provide significant protection against catheter blockage. The failure of Lithostat^®^ to delay catheter blockage in these experiments is most likely due to the burst release nature of drug delivery afforded by this coating, coupled with the continual flow of artificial urine in the bladder models system (Cho *et al*., 2003; Milo *et al*., 2017; Nzakizwanayo *et al*., 2019; Lin *et al*., 2021). Under these conditions, the concentration of Lithostat^®^ achieved in residual bladder urine on coating activation will be continually reduced by urine flow through, and it appears that this does not remain at a sufficient level for long enough to inhibit crystal formation significantly. Although the current coating design containing 5 mg mL^−1^ AHA is expected to result in the generation of concentrations in residual bladder model urine in the range of ∼ 150 – 250µg mL^−1^, which is well above the expected therapeutic range for this drug (indicated as 8 - 30µg mL^−1^ in the prescribing information; (Mission Pharmacal Company, 2023) further optimization of the coating to increase the concentration of AHA delivered may improve performance. The timing of release of AHA may also be a factor, in that pH elevation and crystal formation will already be initiated once coatings have been activated under the conditions tested in our infection models. This could impede the ability of Lithostat^®^ to effectively inhibit further crystal formation when delivered locally *via* our coating. In addition, the recommended use of Lithostat^®^ is as an adjunct to antibiotic therapy (Mission Pharmacal Company, 2023), and combining this with antibacterial agents in coatings should be explored in subsequent studies.

In contrast, the incorporation of ciprofloxacin in our coatings provided significant increases in time to blockage and tripled catheter life span. Fluroquinolones, such as ciprofloxacin, are broad spectrum antibiotics used routinely in UTI therapies (Brown, 2006; Cao *et al*., 2021). According to the National Institute of Health and Care Excellence (NICE) guidelines, ciprofloxacin is recommended as a first choice oral and intravenous antibiotic for patients suffering upper UTI symptoms (National Institute of Health and Care Excellence, 2018). However, recent concerns in regards to the safety profile of ciprofloxacin have been raised, and include reports of disabling and potentially long-lasting side effects (such as tendonitis, arthralgia, and neuropathies associated with paraesthesia, fatigue, memory impairment, depression, and sleep disorders), which have led to new restrictions and precautions in the use of ciprofloxacin (Zacchè *et al*., 2015). As such, the potential for more targeted localized delivery of these agents in response to infection could help prevent problematic side effects, and is also an important feature of strategies aimed at reducing the spread of antibiotic resistance.

Our results are congruent with those of Zhou and colleagues, who also described the localized delivery of ciprofloxacin *via* an infection-responsive catheter coating (Zhou *et al*., 2018). As in our experiments, the coating-mediated delivery of ciprofloxacin was also able to provide significant increases in time to *P. mirabilis* mediated catheter blockage (Zhou *et al*., 2018). However, despite reporting comparable blockage time for uncoated catheters, the increased time to blockage afforded by coatings tested in this study (up to 52.5 h for coatings with ciprofloxacin HCl alone) was notably greater than the increases attained by Zhou *et al*., (2018) (up to 20 h). Incorporation of CF dye in our coating along with ciprofloxacin HCl was able to provide ∼ 79 h advanced warning of catheter blockage, also considerably more than ∼ 28 h (based on initial colour change indicative of pH elevation) reported for analogous coatings by Zhou *et al*., (2018).

Although similar concentrations of ciprofloxacin HCl were incorporated into our coatings and those of Zhou *et al*., (5 and 4.5 mg mL^−1^, respectively), the differences in coating performance are likely attributable to variation in coating designs and the resulting release profiles afforded. The encapsulation of ciprofloxacin HCl into vesicles is reported to provide a slower sustained release in coatings developed by (Zhou *et al*., 2018). While there are potentially clear advantages to a slow sustained release, and such innovations may allow drugs such as Lithostat^®^ to work more effectively when delivered locally *via* infection-responsive coatings, the delivery of antimicrobials to inhibit biofilm formation may work less well in this approach. In contrast, the more rapid burst release expected from our coating design would be expected to expose *P. mirabilis* cells to high initial concentrations of ciprofloxacin HCl at early stages of biofilm formation. These differences in release profile could have contributed to the increased catheter lifespan observed in our coating system.

This hypothesis is congruent with previous studies of ciprofloxacin susceptibility in *P. mirabilis* crystalline biofilms, which demonstrated key roles of biomineralization in protecting cells from this antibiotic and preventing penetration into the biofilm (Li *et al*., 2016). As such a greater exposure to ciprofloxacin HCl at earlier stages of biofilm formation, when susceptibility is higher, would be expected to have a more pronounced impact. Alternatively, the acidic nature of ciprofloxacin could in itself be a factor if its release also serves to temporarily buffer urinary pH and further delay crystal formation, and this mechanism would also be expected to be more pronounced in a burst release system. In addition, anti-urease activity has been reported by ciprofloxacin (and other fluroquinolones) due to their carboxylic group interacting with the nickel atoms present at the active site of the urease enzyme (Kafarski and Talma, 2018), which may also play a role in the increase in blockage times observed.

However, it should be noted that in both our study and that of Zhou *et al*., the elimination of *P. mirabilis* from the bladder model system was not achieved, and cell population had recovered by the time catheters blocked (Zhou *et al*., 2018). Although not statistically significant, the recovery of bacterial populations was potentially more pronounced for coatings containing ciprofloxacin and CF dye in our experiments compared to ciprofloxacin alone (Figure. 3). This most likely reflects the longer time taken for catheters to block in models with ciprofloxacin HCl and CF dye-coated catheters (average of ∼ 14 h longer compared to models with ciprofloxacin only coatings). This increased time to blockage is most likely due to the synergistic effects of ciprofloxacin and CF dye, but will also result in greater dilution of released drugs prior to blockage due to continual urine flow through the system. This dilution effect may result in greater recovery of the bacterial population before the catheters block.

A further notable result of this study is that ‘empty’ coatings devoid of any cargo molecules, as well as those containing the CF dye alone, were able to provide significant increases in time to catheter blockage compared to uncoated controls. This is in contrast to our previous studies, where empty coatings did not impact time to blockage compared to uncoated catheters (Milo *et al*., 2016). In addition, we did not previously observe any inhibitory impact of the CF dye alone on blockage times, although its effect on *P. mirabilis* growth was not evaluated (Milo *et al*., 2016). Furthermore, the current coating design has been modified and optimized and these changes are likely responsible for the impact of empty coatings on blockage time. This likely stems from a mechanical disruption of biofilm formation when coatings are activated. The dissolution of the upper Eudragit S 100^®^ layer and the subsequent detachment and degradation of coating (as observed *via* SEM), should serve to also remove biofilms that have begun to form on these surfaces and around the catheter eyelet. The eyelet in particular is a primary site of blockage and disruption of biofilms that begin to form in this region would be expected to have a notable impact on overall time to blockage (Stickler *et al*., 1999; Stickler, 2008; Stickler and Feneley, 2010; Holling *et al*., 2014).

These observations may point to additional attributes of the infection responsive coatings that could be optimized and exploited to control biofilm formation on urinary catheters. This aspect of our coating technology may be particularly relevant in the context of antibiotic resistance. For example, although ciprofloxacin release would not be expected to provide protection against resistant strains of *P. mirabilis*, the physical effects of coating degradation on biofilm formation should be pertinent regardless of the antibiotic resistance profile of infecting *P. mirabilis* strains. In this context, the incorporation of other non-antibiotic payloads into coatings capable of inhibiting bacterial growth or biofilm formation could also be explored to counter potential issues with antibiotic resistance. For example, the drugs thioridazine and fluoxetine have previously been reported to reduce *P. mirabilis* biofilm formation through efflux inhibition (Nzakizwanayo *et al*., 2017), whilst, the use of novel antimicrobial peptides to control bacterial infection and encrustation on ureteral stents has recently been described (Yao *et al*., 2022).

Overall, this study has demonstrated the potential for theranostic, infection-responsive coatings to form a promising approach to combat catheter encrustation and blockage. Further studies are required to improve coating performance and production, this includes optimization of the agents incorporated, and understanding parameters such as shelf-life, stability, and biocompatibility. Ultimately, the performance of final coating designs will need to be evaluated in clinical trials to clearly establish efficacy, safety, and benefits to patients. However, this proof-of-concept study shows that the continued development of this coating technology has the potential to address a clear and unmet clinical need, which could provide considerable benefits to many patients.

## Data Availability

The data underlying this article will be shared on reasonable request to the corresponding authors.
